# Regulatory T Cell (Treg): Central Orchestrator of Immune Homeostasis

**DOI:** 10.3390/cells15161462

**Published:** 2026-08-15

**Authors:** Md. Abdus Salam, Md. Yusuf Al-Amin, Kasireddy Sudarshan, Nadia Whalen, Faith Chapman, Campbell Gideon, Annabella Cordovez

**Affiliations:** 1 Department of Microbiology, North Bengal Medical College, Sirajganj 6700, Bangladesh; 2 Department of Chemistry and Institute for Drug Discovery, Purdue University, West Lafayette, IN 47907, USA; amin50@purdue.edu (M.Y.A.-A.); kasireddysudarshan@gmail.com (K.S.); 3 Interdisciplinary Life Sciences Graduate Program, Purdue University, West Lafayette, IN 47907, USA; 4 Department of Agricultural and Biological Engineering, Purdue University, West Lafayette, IN 47907, USA; nhwhalen@purdue.edu (N.W.); fchapman@purdue.edu (F.C.); cgideon@purdue.edu (C.G.); 5 Department of Biological Sciences, Purdue University, West Lafayette, IN 47907, USA; acordove@purdue.edu

**Keywords:** FOXP3+ regulatory T cells, immune homeostasis, peripheral tolerance, immunosuppressive cytokines, tissue repair, Treg-based therapies

## Abstract

Regulatory T cells (Tregs) play a pivotal role in maintaining immune homeostasis by exerting precise control over immune activation, suppressing excessive responses, and facilitating tissue repair. These specialized CD4+ T cells, characterized by FOXP3 expression, function as key regulators that prevent pathogen-directed immune responses from progressing to deleterious autoimmunity or chronic inflammation. Tregs mediate suppression via secretion of cytokines such as IL-10 and TGF-β, metabolic disruption, and direct modulation of effector immune cells, thereby maintaining equilibrium between protective immunity and peripheral tolerance. Both thymically derived natural Tregs (nTregs) and peripherally induced Tregs (pTregs) exhibit phenotypic plasticity, adapting to diverse inflammatory milieus and tissue microenvironments through an array of suppressive mechanisms that orchestrate immune regulation and facilitate tissue repair. This functional heterogeneity manifests across lymphoid and non-lymphoid tissues, wherein Tregs dynamically adapt to distinct microenvironments to mount tailored responses to infection, tissue injury, and inflammatory insults. Conversely, Tregs may promote disease progression in malignancies and persistent infections by attenuating antitumor and antimicrobial immune effector responses. Treg activity is essential for averting autoimmune pathologies, tempering inflammatory cascades, and fostering tissue regeneration, thereby rendering them indispensable for upholding both systemic and tissue-specific immune homeostasis. Elucidation of Treg immunobiology unveils substantial therapeutic prospects across a diverse array of pathologies; targeted modulation of Treg frequency and functionality offers promise for ameliorating autoimmunity, mitigating transplant rejection, and combating malignancy. This narrative review delineates the multifaceted roles of Tregs in immune homeostasis, elucidates emerging insights into their mechanistic underpinnings, and evaluates prospective applications in next-generation immunotherapeutic interventions.

## 1. Introduction

The 2025 Nobel Prize in Physiology or Medicine was jointly awarded to Mary E. Brunkow, Fred Ramsdell, and Shimon Sakaguchi for their seminal discoveries elucidating the mechanisms whereby the immune system preserves homeostasis by forestalling assaults on self-tissues. Their foundational work delineated the existence and suppressive functions of regulatory T cells (Tregs), a distinct CD4+ T-cell subset that curbs hyperactive immune responses and enforces peripheral tolerance to avert autoimmunity. Tregs operate as a dynamic immune rheostat, tuning the immune response rather than simply switching it off. They restrain excessive inflammation by suppressing overactive effector cells, yet in the right context they also permit controlled activation needed for host defense, tissue repair, and immune homeostasis. Immune homeostasis depends on a specialized T-cell lineage that limits excessive effector activity and safeguard against autoimmunity. Canonically defined by their proficiency in suppressing effector immunity and enforcing self-tolerance, Tregs have solidified their status as master regulators of peripheral immune equilibrium [1,2,3]. Distinguished by constitutive expression of the FOXP3 (Forkhead box protein P3) transcription factor, Tregs function as pivotal guardians, suppressing autoimmune reactivity, constraining pathologic inflammation, and mitigating collateral tissue injury [4,5]. Tregs maintain immune homeostasis through multifaceted suppressive modalities, including secretion of immunosuppressive cytokines such as IL-10, IL-35, and TGF-β (transforming growth factor beta); direct cell–cell contact-mediated inhibition; and subversion of antigen-presenting cells (APCs) via inhibitory receptors like CTLA-4 (Cytotoxic T-lymphocyte–associated protein 4) and PD-1 (Programmed cell death protein 1) [6,7,8]. These suppressive mechanisms are indispensable not only for curtailing hyperactive immune responses but also for calibrating the magnitude and qualitative profile of immunoreactivity toward both self- and foreign antigens. Beyond autoimmunity suppression, Tregs profoundly influence diverse physiologic and pathologic paradigms, encompassing orchestration of tissue repair, attenuation of infection-associated immunopathology, and modulation of effector immunity within the tumor microenvironment (TME) [9,10,11]. This duality, as suppressors of pathologic immunity and enablers of tumor immune escape, underscores the intricate biology of Tregs and the precarious equilibrium that underpins immune homeostasis [1]. Contemporary advances have substantially expanded insights into Treg biology, revealing functions that transcend canonical immunosuppression to encompass tissue-specific adaptations, including metabolic regulation, orchestration of tissue remodeling, and reciprocal crosstalk with stromal compartments [10,12,13]. This paradigmatic shift illuminates the pleiotropic roles of Tregs in physiologic homeostasis and pathologic progression. Delineating mechanisms of Treg ontogeny, effector functions, and plasticity heralds transformative therapeutic avenues for autoimmune diseases, allograft rejection, allergic diatheses, and oncologic malignancies [14,15].

## 2. Biology of Tregs

Regulatory T cells comprise a specialized CD4+ T lymphocyte subset, phenotypically defined by high FOXP3 transcription factor expression, elevated levels of CD25 (which is the alpha chain of the interleukin-2 receptor), and diminished or absent levels of CD127 (the alpha chain of the interleukin-7 receptor) [16]. Distinguished by constitutive FOXP3 expression, Tregs function as pivotal guardians, suppressing autoimmune reactivity, constraining pathologic inflammation, and mitigating tissue injury [3]. Early mouse models demonstrated that the lack or dysfunction of Tregs leads to catastrophic autoimmune pathology and multiorgan inflammation, highlighting their nonredundant role in safeguarding immune equilibrium [17]. In humans, congenital deficiencies in FOXP3 result in severe autoimmune syndromes, further reinforcing the critical nature of Tregs in immune regulation [18].

### 2.1. Developmental Ontology and Phenotypes of Tregs

**Figure 1:**
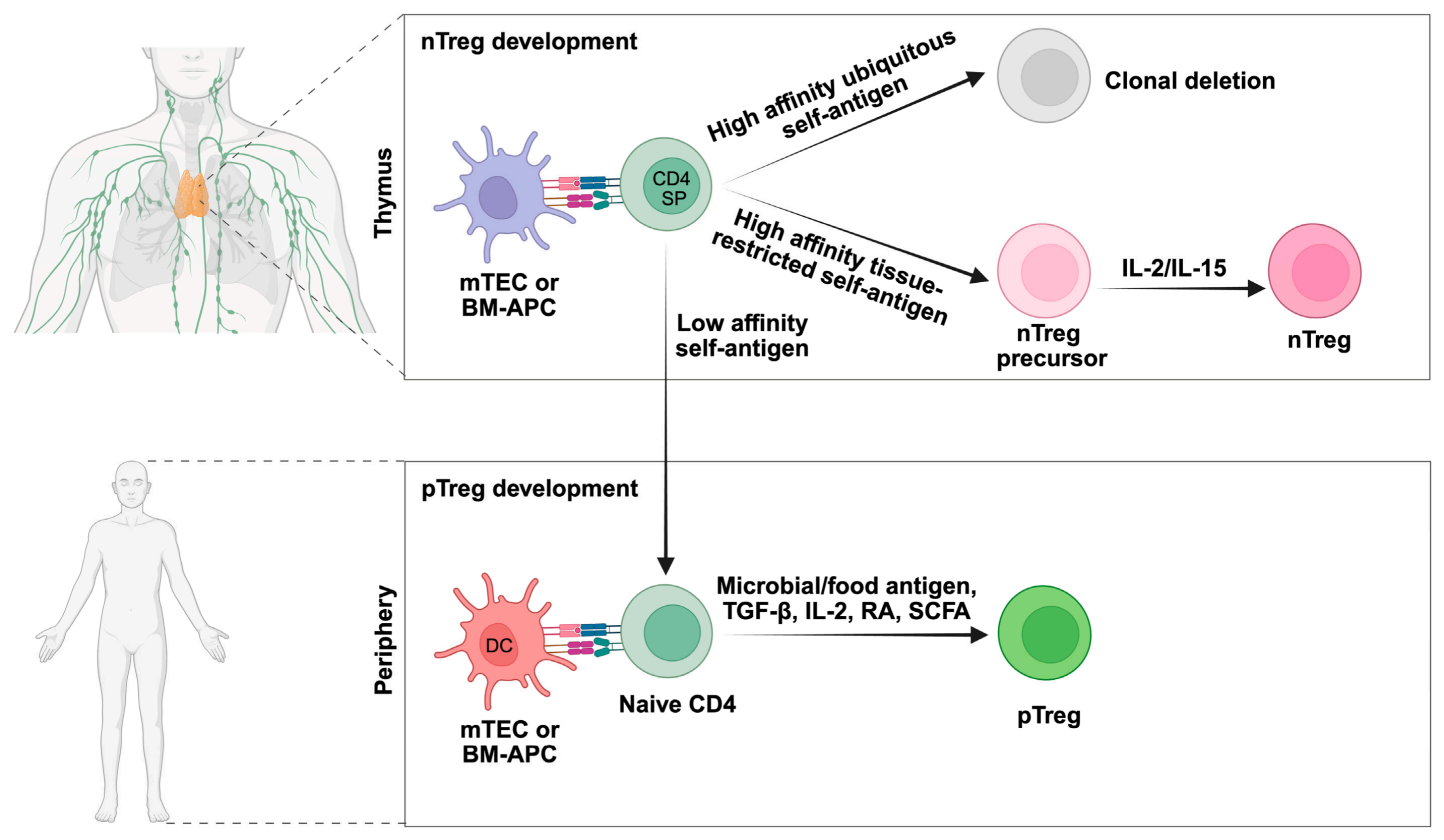
Schematic diagram of Tregs development: natural Tregs (nTregs) and peripheral Tregs (pTregs): nTregs originate in the thymus through a two-step mechanism. In the first step, high-affinity tissue-specific self-antigens, presented by medullary thymic epithelial cells (mTEC) or bone-marrow (BM) derived antigen-presenting cells (APC), direct single-positive (SP) T cells (a mature thymocyte that expresses CD4 only) towards the Treg lineage. In the second step, cytokines such as IL-2 or IL-15 help these precursor cells become fully mature nTregs. On the other hand, pTregs are generated in peripheral tissues by environmental antigens such as microbial or food antigens presented by mucosal tissue-resident dendritic cells (DCs). The transformation of pTregs is further promoted in immunosuppressive environments rich in TGF-β, retinoic acids (RAs), and short chain fatty acids (SCFAs). Created in BioRender. Amin, M. Y. A. https://BioRender.com/p3b0nsf (accessed on 18 July 2026).

Treg developmental ontogeny manifests a fundamental duality, delineating thymically derived natural Tregs (nTregs) from peripherally induced Tregs (pTregs), that act synergistically to maintain peripheral immunological tolerance. Thymic selection imposes self-tolerance via nTreg cells, comprising precommitted subsets shaped by high-avidity TCR:self-peptide:MHC interactions; these derive from discrete progenitor pools, collectively yielding a polyclonal, self-reactive TCR repertoire [19]. nTregs display constitutive, stable FOXP3 expression, exerting a cardinal role in sustaining systemic self-tolerance and forestalling autoimmunity toward circulating and lymphoid self-antigens [20]. Epigenetic regulation is also important for Treg stability, and demethylation of the Treg-specific demethylated region (TSDR) within the FOXP3 locus is a key feature of stable, committed Tregs. This open chromatin state helps maintain durable FOXP3 expression and supports suppressive function, whereas incomplete TSDR demethylation is associated with less stable Treg identity [21]. Peripherally induced regulatory T cells (pTregs or iTregs) fulfill non-redundant functions in enforcing tolerance to environmental antigens and extended-self constituents, including commensal microbiota and alimentary antigens, predominantly within mucosal sites such as the intestine [22]. In contrast to thymically derived nTregs, which predominantly enforce self-tolerance to autoantigens, pTregs arise extrathymically from naïve CD4+ T cells upon encountering extrinsic antigens, particularly under subdominant TGF-β and IL-2 signaling [23] (Figure 1). Postnatally, pTregs differentiate at mucosal barriers, driven by commensal-derived metabolites like retinoic acid and specialized APCs such as CD101+ dendritic cells. In vitro, iTregs, functionally analogous to pTregs are induced via targeted cytokine milieus from naïve precursors [24]. Together, nTregs provide foundational immune tolerance, while pTregs adaptively respond to new antigens that arise in peripheral tissues, controlling inflammation and maintaining immune homeostasis in those environments [25,26].

### 2.2. Molecular Markers

**Figure 2:**
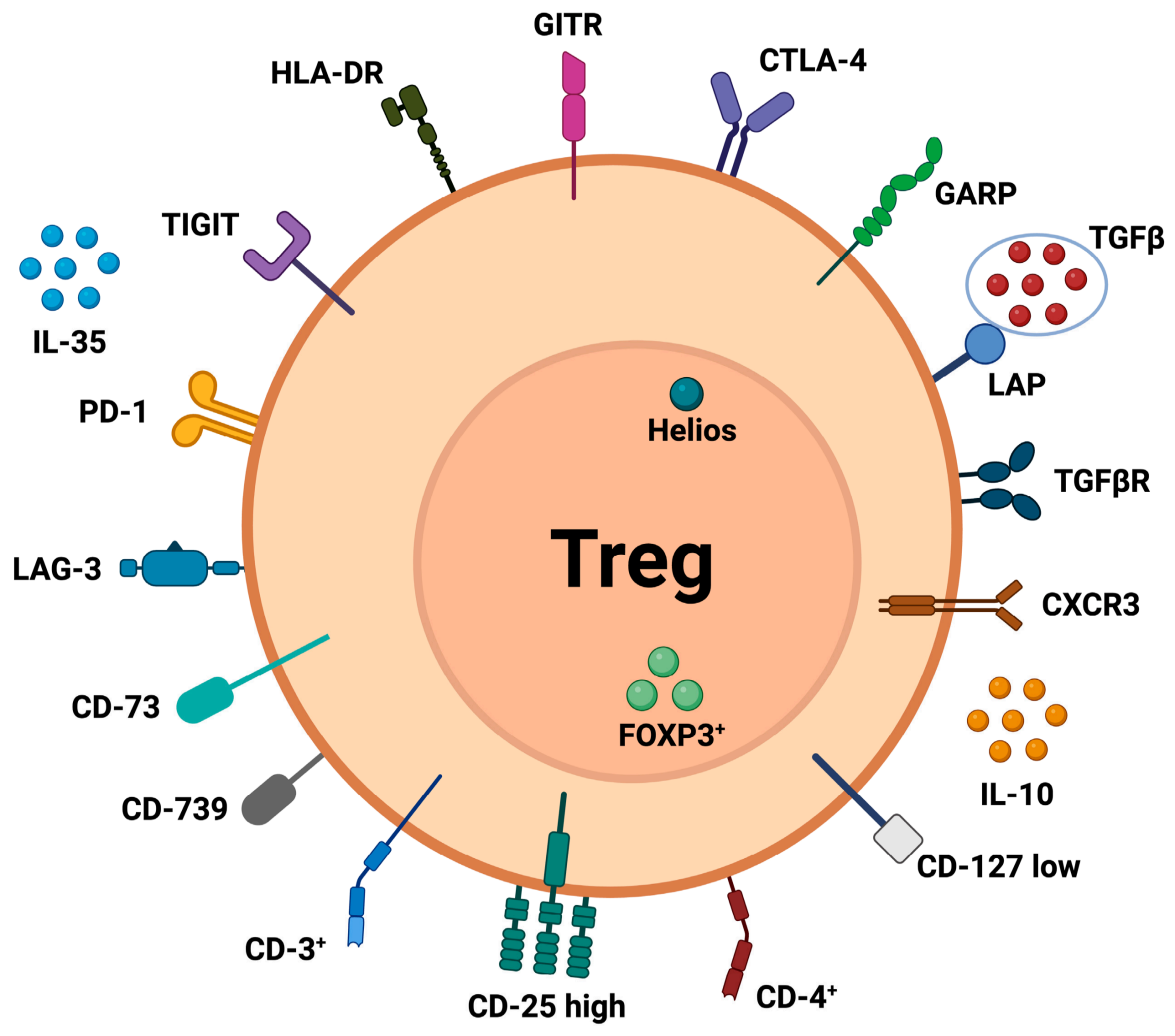
Markers of regulatory T cells (recognized as FOXP3+ CD4^+^CD25^high^CD127^low^/^−^): CD25 high, CD127 low: Activation and lineage markers. CTLA-4, PD-1, LAG-3: Checkpoint inhibitors for suppression. GITR, HLA-DR: Co-stimulatory and activation indicators. CD39, CD73: Ectoenzymes generating adenosine. Helios, LAP, GARP: Stability and TGF-β latency markers. CD3, CD4+: Basic T cell identifiers. TGF-β, IL-10, IL-35: Key suppressive mediators. CXCR3: Migration receptor. Created in BioRender. Amin, M. Y. A. https://BioRender.com/uenznfv (accessed on 18 July 2026).

Regulatory T cells are delineated by a characteristic constellation of surface and intracellular markers, encompassing CD4+CD25highCD127lowFOXP3+ expression that discriminates them from conventional T cells, facilitating interrogation of their suppressive capacity in experimental and clinical paradigms. The most critical marker is the transcription factor FOXP3, which is essential for Treg development, lineage specification, and suppressive function. The FOXP3 transcription factor, via its forkhead (FKH) and leucine-zipper (Zip) domains, binds cognate DNA motifs to orchestrate transcriptional regulation of target genes [27,28]. These DNA-protein interactions empower FOXP3 to transactivate or repress loci encoding suppressive effectors, thereby endowing Tregs with the capacity to avert dysregulated immunity and sustain homeostasis. FOXP3 is indispensable for self-tolerance, as evidenced by its ablation precipitating fulminant autoimmune phenotypes, such as IPEX (Immunodysregulation polyendocrinopathy enteropathy X-linked) syndrome in humans [5,17]. Tregs further express CD4, high CD25 (IL-2 receptor α) and low or absent CD127 (IL-7 receptor α). The CD4^+^CD25^high^CD127^low^/^−^ phenotype constitutes the gold standard for ex vivo identification of viable human Tregs [29]. Additional molecules frequently associated with Treg identification and function include CTLA-4, GITR (Glucocorticoid-induced TNFR-related protein), LAG-3 (Lymphocyte activation gene-3), TIGIT (T cell immunoreceptor with Ig and ITIM domains), LAP (latency-associated peptide), GARP (glycoprotein A repetitions predominant), CD39 and CD73 (enzymes involved in immunosuppressive adenosine production), PD-1 and Helios (particularly for nTregs) [30] (Figure 2). Distinctive molecular signatures enable discrimination of suppressive (functionally competent) Treg subsets from non-suppressive (dysfunctional or plastic) counterparts, particularly within pathologic milieus [31]. Constitutive high FOXP3 expression remains the preeminent marker of suppressive Treg potency, frequently conjoined with ancillary surface/intracellular determinants. Collectively, stable FOXP3^high^, CD25^high^, CD127^low^, and expression of Helios, TIGIT, CTLA-4, and the CD39/CD73 ectonucleotidase axis robustly denote suppressive Treg subsets. Conversely, ablation or dysregulated expression of these markers heralds non-suppressive or effector-destined Tregs [17,32].

### 2.3. Critical Functions of Tregs in Modulating Immunity

Tregs exert pivotal suppressive functions in immune responses by inhibiting effector CD4^+^ and CD8^+^ T lymphocytes, alongside innate immune populations, thereby maintaining self-tolerance, resolving inflammation, and supporting tissue repair. They are essential for upholding self-tolerance, averting immunopathology, and fine-tuning immune responses to infections and malignancies, positioning them as key mediators of immune homeostasis and prime candidates for therapeutic intervention [33]. Both nTregs and pTregs cooperate synergistically to sustain peripheral immune tolerance against exogenous antigens [19,22]. Tregs maintain immune homeostasis, suppress hyperactive responses, and constrain autoimmunity through following diverse, context-dependent mechanisms tailored to tissue microenvironments and immune challenges [10,14,33].

- Tregs avert autoimmunity by enforcing peripheral tolerance through multifaceted suppressive mechanisms. These encompass secretion of anti-inflammatory cytokines (e.g., IL-10, TGF-β, IL-35); metabolic disruption via cytokine sequestration (notably IL-2), adenosine generation, and cAMP transfer; direct cytolysis of effector cells mediated by granzymes and perforin; and APC modulation through surface receptors (CTLA-4, LAG-3) that impair T cell priming.
- Tregs further constrain chronic inflammation and uphold immune homeostasis, thereby mitigating the incidence of allergic disorders, autoimmunity, and dysregulated inflammatory cascades.
- In pathological states such as malignancy, heightened Treg activity dampens anti-tumor immunity and promotes tumor progression, thereby posing significant challenges to immunotherapeutic strategies.

## 3. Mechanisms of Immune Suppression by Tregs

**Figure 3:**
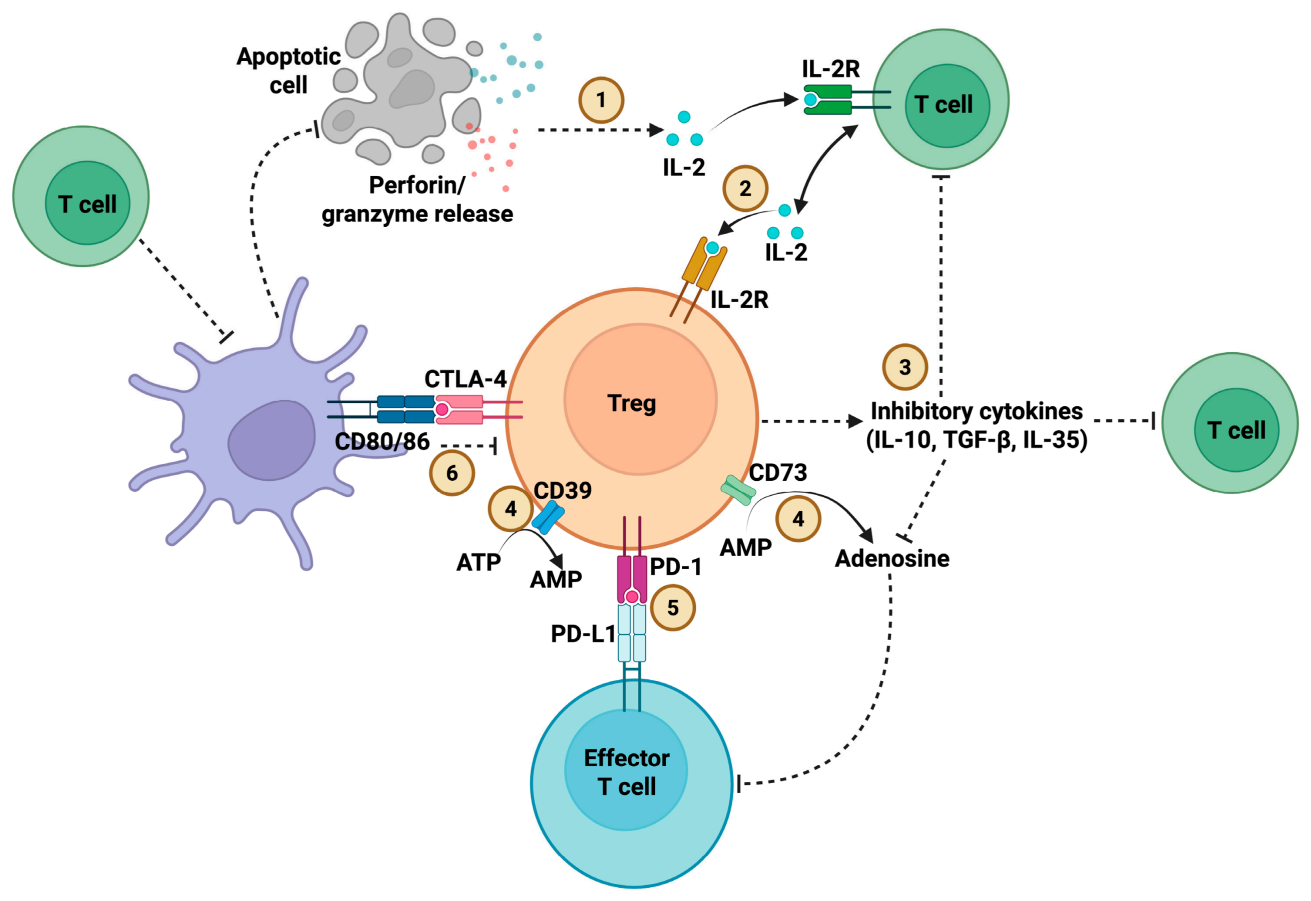
Schematic illustrating six key immunosuppressive pathways mediated by Tregs: (1) Apoptotic cell clearance via perforin/granzyme induces IL-2 signaling through IL-2R. (2) Treg CD25 (IL-2Rα) expression sequesters IL-2. (3) Tregs secrete immunosuppressive cytokines (IL-10, TGF-β, IL-35) to dampen immune activity. (4) CD39/CD73 ectonucleotidases generate immunosuppressive adenosine from ATP. (5) PD-1/PD-L1 checkpoint inhibition. (6) CTLA-4 outcompetes CD28 for CD80/86 on APCs. Dashed lines indicate inhibitory pathways; solid lines indicate activating signals. Created in BioRender. Amin, M. Y. A. https://BioRender.com/qx7mhss (accessed on 18 July 2026).

Tregs employ multifaceted, context-dependent mechanisms to suppress immune responses, targeting effector T cells, APCs, and innate cells while promoting tolerance. They secrete suppressive cytokines, including IL-10, TGF-β, and IL-35, which attenuate inflammation and inhibit the activation and proliferation of effector T cells, skew APC function toward tolerance, and dampen Th1/Th17 differentiation and innate immune cells. Tregs kill activated effector T cells via perforin/granzyme-induced apoptosis, thereby removing these IL-2–consuming cells and increasing local IL-2 availability for themselves. High constitutive expression of the high-affinity IL-2 receptor (especially CD25) allows Tregs to capture this IL-2, enhancing IL-2R–STAT5 signaling that maintains Foxp3 expression and suppressive function. Thus, perforin/granzyme-mediated clearance of effector cells indirectly boosts IL-2 signaling in Tregs and reinforces Treg-mediated immunosuppression. Tregs further shape the immune milieu by sequestering IL-2, thereby depriving effector T cells of essential growth signals, and by generating adenosine via CD39/CD73 ectoenzymatic activity to potently suppress immune cell function [3,13,34]. Contact-dependent mechanisms employ CTLA-4, LAG-3, and TIGIT to disrupt costimulatory signaling and confer tolerogenic phenotypes on dendritic cells, whereas cytolytic pathways leverage granzyme and perforin to trigger apoptosis in target cells by inducing caspase-independent apoptosis in effector T cells, NK cells, and B cells. This perforin-dependent killing targets activated immune cells without bystander damage [35] (Figure 3). Thus, through multifaceted mechanisms, Tregs are indispensable for upholding self-tolerance, minimizing collateral tissue damage during immune responses, and perpetuating immune homeostasis. Their homeostatic functions are foundational to establishing a balanced, efficacious immune system. Emerging evidence underscores that Treg mechanisms and functions are sculpted by tissue microenvironments through dynamic interactions with stromal and parenchymal cells [9,36].

## 4. Tissue-Specific Adaptations and Functions of Tregs

Tregs adapt to specific tissues beyond lymphoid organs to suppress immunity and support homeostasis, repair, and regeneration. Recent technological advances have illuminated substantial heterogeneity among Tregs, especially within different tissue niches [12,37]. Treg subsets are transcriptionally and epigenetically programmed to adapt to their tissue environment and functional demands. Tissue-resident Tregs and effector Tregs acquire activated, highly suppressive states, whereas tumor-associated Tregs are further shaped by the immunosuppressive tumor microenvironment to sustain potent inhibitory activity. In the intestine, microbiota-associated Tregs develop distinct programs that promote mucosal tolerance, and metabolically specialized Tregs adjust their gene expression and epigenetic landscape in response to local nutrient and cytokine cues, enabling context-specific immune regulation. Recent single-cell RNA sequencing, spatial transcriptomics, and multi-omics studies have revealed that Tregs are highly heterogeneous, comprising distinct tissue- and state-specific subsets with specialized transcriptional and functional programs. These approaches have also shown that Treg identity and suppressive activity are shaped by local microenvironmental cues, highlighting the importance of context-dependent regulation in maintaining immune homeostasis [15]. Tissue-resident Tregs undergo a two-step differentiation: partial adaptation in secondary lymphoid organs via TCR-specific priming, followed by full maturation in target tissues influenced by local cues like cytokines and metabolites. Key examples of tissue-resident Tregs and their principal functions are outlined below [38].

- Intestinal Tregs: In the colon, peripherally induced Tregs produce IL-10 to enforce microbiota tolerance, featuring RORγt+ (Retinoid-related orphan receptor gamma t) subsets for pathobiont control and GATA3+ (GATA Binding Protein 3) populations that secrete amphiregulin (Areg) during epithelial damage. Small intestinal counterparts, shaped by TGF-β, retinoic acid, and microbial short-chain fatty acids from dendritic cells, prioritize dietary antigen tolerance.
- Pulmonary Tregs: Lung-resident Tregs promote resolution of acute lung injury or viral infection through Areg and keratinocyte growth factor secretion, fostering epithelial repair while curbing excessive inflammation via IL-10.
- Musculoskeletal and cutaneous Tregs: In skeletal muscle, Tregs engage satellite cells via Areg to accelerate regeneration and attenuate fibrosis post-injury. Dermal Tregs similarly support hair follicle cycling and scarless wound remodeling.
- Central nervous system Tregs: Specialized Tregs support neuronal survival and modulate microglial activation, contributing to CNS homeostasis and potentially participating in neurorepair.
- Adipose tissue Tregs: Visceral adipose tissue (VAT)-enriched Tregs upregulate Peroxisome proliferator-activated receptor gamma (PPARγ) and lipid metabolism genes [e.g., Diacylglycerol acyltransferase type 1 (Dgat1), Global deletion of CTα (Pcyt1a)], enabling them to mitigate obesity-associated inflammation and maintain metabolic balance Tregs in visceral adipose depots regulate inflammation and metabolic homeostasis, impacting systemic insulin sensitivity.

These functional adaptations underscore the remarkable plasticity of Tregs, enabling them to sense local environmental cues and tailor their effector programs to execute specialized immunoregulatory functions within distinct tissue niches.

## 5. Non-Immunosuppressive Roles of Tregs

Beyond their canonical immunosuppressive functions, Tregs play pivotal roles in tissue repair and regeneration through direct secretion of growth factors such as amphiregulin. At sites of injury, particularly in the lung, muscle, and skin, Tregs elaborate amphiregulin to engage local stem or progenitor cells, thereby driving restorative proliferation and functional recovery [9,39]. Tregs also orchestrate local metabolic homeostasis across diverse tissues, notably in visceral adipose tissue where they safeguard energy balance. Through PPARγ-dependent mechanisms, they modulate lipid metabolism and enhance insulin sensitivity, thereby adapting metabolically to suppress inflammation associated with insulin resistance [40,41]. In addition to modulating immune responses, Tregs exert profound influence on tissue architecture by engaging stromal elements, including fibroblasts and epithelial cells, while directly orchestrating wound healing dynamics [37,42]. Further, Tregs sustain quiescence and functionality of tissue-resident stem cells in the skin, intestine, and bone marrow by mitigating oxidative stress and bolstering regenerative potential [9,43]. These context-dependent, tissue-adaptive functions have emerged as central to the expansive physiological repertoire of Tregs, rendering them indispensable not only for immune tolerance but also for the preservation and repair of diverse organs. Thus, Tregs orchestrate a unified program of immune quiescence and tissue homeostasis, affirming their critical role in health maintenance and disease resolution [44,45].

## 6. Treg Plasticity and Stability

Regulatory T cells exhibit extraordinary plasticity and stability, hallmarks of their efficacy within intricate immune milieus. Treg plasticity helps them adapt to tissue-specific cues, allowing them to support repair, maintain local homeostasis, and tailor suppression to distinct inflammatory environments. However, when this plasticity becomes dysregulated under chronic inflammation, Tregs may lose FOXP3 stability and acquire pro-inflammatory features such as IL-17 or IFN-γ production, contributing to tissue damage and disease progression [37]. This plasticity manifests as their capacity to adopt phenotypic features of diverse T helper (Th) subsets, such as T-bet for Th1-like, GATA3 for Th2-like, Bcl6 for Tfh-like, or RORγt for Th17-like differentiation, while predominantly retaining FOXP3 expression and suppressive function. In this broader heterogeneity, follicular regulatory T (Tfr) cells represent a specialized subset that localizes to germinal centers and restrains B-cell and antibody responses, whereas type 1 regulatory T (Tr1) cells are FOXP3-independent regulatory cells characterized by high IL-10 production and potent anti-inflammatory activity. Such adaptability empowers Tregs to counter varied inflammatory contexts through integration of environmental cues, cytokines, and tissue-derived signals, thereby optimizing their context-specific immunosuppression [46,47].

Stability, by contrast, denotes the enduring FOXP3 expression and preservation of Treg-specific epigenetic landscapes, such as CNS2/TSDR demethylation, which safeguard lineage fidelity and suppressive potency. Cytokines like IL-2 reinforce this stable suppressive state, whereas inflammatory signals such as IL-6 and IL-12 can weaken FOXP3 expression, increase plasticity, and promote loss of suppressive identity [48]. This steadfastness minimizes the risk of FOXP3 loss or transdifferentiation into pro-inflammatory effectors, thereby ensuring robust Treg-mediated immunosuppression within the immune repertoire. In autoimmune diseases and chronic inflammation, Treg stability is frequently destabilized, resulting in FOXP3 downregulation alongside upregulated expression of pro-inflammatory factors such as RORγt and cytokines including IL-17 and IFN-γ. Excessive Treg instability precipitates suppressive dysfunction or outright acquisition of pathogenic traits, thereby exacerbating autoimmunity. Pivotal regulators of this plasticity-stability axis encompass cytokines (e.g., IL-6, IL-12), metabolic cascades (glycolysis, mTOR, PI3K/AKT signaling), and epigenetic remodeling [47,49,50].

## 7. Pathophysiological Roles of Tregs

Regulatory T cells have emerged as pivotal modulators in human pathology through their potent orchestration of immune homeostasis. Functioning as double-edged swords, these cells temper excessive or aberrant immunity to avert tissue damage and autoimmunity, yet their suppressive and tissue-homeostatic programs can paradoxically exacerbate disease progression in contexts such as malignancy, chronic infection, and fibrosis by blunting protective effector responses. Implicated across diverse pathologies including autoimmunity, persistent infections, allergies, and neoplasia, the delicate equilibrium of Treg activity ultimately dictates outcomes ranging from tolerance and protection to pathological dominance [21,51].

### 7.1. Tregs and Autoimmune Diseases

Regulatory T cells are indispensable for upholding immune tolerance and forestalling autoimmunity through suppression of self-reactive lymphocytes. They achieve this via cell-contact-dependent mechanisms, secretion of anti-inflammatory cytokines such as IL-10 and TGF-β, and modulation of APC activity. In autoimmune diseases including type 1 diabetes, multiple sclerosis, and systemic lupus erythematosus, deficiencies in Treg frequency, stability, or suppressive capacity precipitate tolerance breakdown [3,17,52]. Therapeutically, strategies to expand Treg populations or augment their function hold substantial promise for restoring tolerance and attenuating progression across diverse autoimmune disorders.

Key therapeutic strategies for autoimmune diseases are delineated below.

- Low-dose IL-2 therapy: Tregs respond preferentially to low concentrations of IL-2, owing to their high-affinity IL-2 receptor expression. Clinical trials have demonstrated efficacy in mitigating disease activity in type 1 diabetes, graft-versus-host disease (GvHD), alopecia areata, and autoimmune hepatitis [33,34].
- Adoptive transfer of Treg cell: Infusion of polyclonal or antigen-specific Tregs, frequently expanded ex vivo, represents a burgeoning cell therapy modality under evaluation in clinical trials for diverse autoimmune disorders. This strategy aims to re-establish immune tolerance and restrain dysregulated autoimmunity through direct augmentation of Treg frequency and functionality in vivo. Chimeric antigen receptor (CAR) engineering of regulatory T cells enables precise targeting of autoantigens, thereby potentiating therapeutic efficacy [21,53,54,55].
- Pharmacologic agents: Disease-modifying antirheumatic drugs (DMARDs; e.g., methotrexate), mTOR inhibitors (e.g., rapamycin), and TNF-α antagonists, foster Treg induction, expansion, and FOXP3 upregulation in vivo while enhancing their suppressive function. Select biologics concurrently augment Treg populations and attenuate effector T cell responses [56].
- Other cytokines and ligands: Agents such as IL-10, TNF receptor 2 (TNFR2) agonists, and FMS-like tyrosine kinase 3 (FLT3) ligands promote Treg induction, expansion, and restoration of immune homeostasis [21].
- Gene editing: CRISPR/Cas9-mediated gene editing and analogous platforms are under active investigation to generate engineered Tregs with augmented stability, antigen specificity, and immunosuppressive potency. These approaches target key loci such as FOXP3 enhancers or lineage-stability cassettes to mitigate plasticity-related dysfunction while conferring precise autoreactivity, holding transformative potential for personalized immunotherapy in refractory autoimmunity. Preclinical models demonstrate sustained engraftment and superior tolerance induction, paving the way for clinical translation [38,57].
- Combination therapies: Therapies integrating Treg interventions with immunomodulatory drugs or complementary cellular approaches seek to amplify immune regulatory efficacy. Such synergistic strategies, exemplified by low-dose IL-2 paired with rapamycin or Treg transfer alongside anti-CD3 monoclonal antibodies leverage orthogonal mechanisms to bolster Treg stability, expansion, and suppressive function while countering effector T cell hyperactivity. Preclinical and early-phase trials indicate enhanced tolerance induction and disease amelioration, particularly in refractory settings like graft-versus-host disease and systemic lupus erythematosus, heralding a paradigm shift toward multimodal precision immunomodulation Approaches combining Treg therapy with immunomodulatory drugs or other cell therapies aim to enhance the efficacy of immune regulation [58,59].

Collectively, these strategies facilitate tolerance restoration, attenuate immunopathology, and diminish reliance on nonspecific immunosuppression for autoimmune disease management.

### 7.2. Tregs and Allergic Diseases

Regulatory T cells are central to immune homeostasis because they restrain excessive effector responses, maintain peripheral tolerance, and prevent harmless environmental antigens from being misclassified as pathogenic. Emerging evidence indicates that Tregs are not a fixed population but instead display context-dependent instability and plasticity, with the capacity to transition between functional and dysfunctional states. Understanding these dynamic regulatory states across pathological settings is therefore of considerable importance [60]. In allergic disease, this homeostatic role is particularly critical. In healthy individuals, allergen-specific Tregs preserve tolerance at mucosal and epithelial barriers, including the airways, skin, and gut, through the production of regulatory cytokines such as IL-10 and TGF-β, contact-dependent suppressive mechanisms, and the promotion of noninflammatory antibody class switching. By dampening dendritic-cell activation, limiting Th2 polarization, reducing eosinophilic inflammation, and constraining IgE-mediated responses, Tregs serve as a pivotal checkpoint in the initiation and persistence of allergic sensitization [61]. In contrast, allergic disorders are frequently associated with both quantitative and functional Treg defects, including reduced cell numbers, impaired suppressive capacity, unstable Foxp3 expression, and exposure to a proinflammatory milieu that weakens regulatory control and may promote pathogenic reprogramming. Such abnormalities have been reported in allergic rhinitis, asthma, food allergy, and atopic dermatitis, supporting the view that allergy reflects not only exaggerated effector immunity but also defective immune regulation [62,63]. Treg development and function are shaped by cytokine signals, metabolic and nutrient cues, tolerogenic antigen-presenting cells, tissue-derived metabolites such as retinoic acid, co-stimulatory and co-inhibitory pathways, and the microbiota, all of which influence Treg stability and suppressive capacity. Within this framework, the microbiota–Treg axis represents a bidirectional regulatory circuit in which commensal organisms and their metabolites promote mucosal tolerance, whereas disruption of this interaction favors allergic sensitization. In particular, microbial products such as short-chain fatty acids support Foxp3+ Treg differentiation and stability, promote tolerogenic dendritic-cell programming, and limit Th2-skewed inflammation at barrier surfaces. Early-life antibiotic exposure, dietary perturbations, and dysbiosis may therefore weaken Treg-mediated control and increase susceptibility to atopic dermatitis, food allergy, and allergic asthma, whereas a diverse microbiome enriched in metabolite-producing taxa helps preserve mucosal homeostasis and restrain IgE-driven responses [64,65].

Importantly, successful allergen immunotherapy can restore Treg frequency and function, underscoring the therapeutic relevance of Treg biology in allergic disease. Collectively, these findings suggest that Tregs are not passive suppressors but active coordinators of immune equilibrium, with their stability, tissue adaptation, and antigen specificity helping determine whether allergen exposure leads to tolerance or chronic inflammation [66].

### 7.3. Tumor-Infiltrating Tregs and Cancers

Tumor-infiltrating Tregs play a major role in suppression of anti-tumor immune responses within the tumor microenvironment (TME), often facilitating cancer progression and immune evasion. These Tregs accumulate within tumor sites, exhibiting heightened suppressive function that enables cancer cells to evade immune surveillance and resist clearance by cytotoxic lymphocytes. They mediate suppression through secretion of immunosuppressive cytokines including IL-10 and TGF-β, expression of checkpoint molecules such as CTLA-4, and modulation of dendritic cells alongside other immune populations. Tumor-infiltrating Tregs are associated with unfavorable prognosis across multiple malignancies, including breast cancer, lung cancer, and melanoma [67].

Key features of tumor-infiltrating Tregs

- Regulatory T cells are actively recruited and enriched within the tumor microenvironment and peripheral blood of cancer patients, often representing a substantially higher proportion of T cells in tumor tissue compared to normal tissue [68].
- These cells inhibit the function of cytotoxic CD8+ T cells, natural killer (NK) cells, and antigen-presenting cells, thereby attenuating antitumor immunity and enabling tumor progression [69].
- Tregs exert suppression via immunosuppressive cytokines (IL-10, TGF-β, IL-35), contact-dependent mechanisms (CTLA-4, LAG-3), metabolic disruption (adenosine generation through CD39/CD73), and direct cytotoxicity (granzyme B/perforin pathways), targeting effector immune cells in the TME [8,70].
- Elevated intratumoral Treg abundance correlates with adverse prognosis and enhanced tumor aggressiveness across diverse malignancies, including breast, lung, gastric, hepatocellular, pancreatic, and ovarian cancers [13].
- By fostering immunosuppression, Tregs promote immune evasion by tumor cells, thereby facilitating growth, angiogenesis, and metastasis [71].
- High intratumoral FOXP3+ Treg levels are associated with reduced survival and elevated recurrence rates post-therapy in multiple cancers [72].
- Tregs further undermine cancer immunotherapies, including checkpoint inhibitors, by sustaining an immunosuppressive TME [70,73].

Ongoing research and clinical trials are investigating strategies to deplete or functionally inhibit tumor-infiltrating Tregs to potentiate immunotherapies, including checkpoint inhibitors and cancer vaccines. These approaches encompass targeting Treg-specific surface markers (e.g., CD25, CCR4), blocking recruitment pathways into the tumor microenvironment, or pharmacologically modulating their suppressive activity through agents like anti-CTLA-4 antibodies or low-dose cyclophosphamide [74]. Recent studies highlight CCR8^+^, TNFR2^+^, TIGIT^+^, and highly activated effector Tregs as particularly suppressive tumor-infiltrating subsets, making them attractive therapeutic targets in cancer. Current strategies include selective depletion, functional inhibition, blocking Treg trafficking, and combination with immune checkpoint blockade to reduce tumor Treg-mediated immunosuppression while preserving broader immune function [75]. Consequently, deeper insights into Treg biology and selective manipulation offer substantial promise for advancing novel, efficacious anticancer therapies.

## 8. Prospects and Challenges in Treg Targeting

Regulatory T cells offer substantial therapeutic potential across diverse pathologies owing to their distinctive ability to suppress dysregulated or excessive immune responses. CAR-Treg therapy has potential in cancer by enabling engineered regulatory T cells to suppress harmful immune responses in a targeted way, which may help control tumor-associated inflammation and improve treatment settings where immune regulation is needed. Current CAR targets under investigation include CD19, BCMA, CD22, and CD20 in blood cancers, while B7-H3, HER2, EGFRvIII, GD2, and CLDN18.2 are being actively explored in solid tumors due to their frequent tumor-associated expression and therapeutic potential [76]. Recent work showed that FOXP3 engineering reprograms CAR-T cell metabolism, improving persistence, reducing exhaustion, and enhancing long-term antitumor efficacy without inducing Treg-like immunosuppression [77]. Strategies to harness Treg function are under active investigation for treating autoimmune diseases, preventing allograft rejection, and mitigating inflammation in allergic and chronic inflammatory conditions [35,78]. Adoptive CAR-Treg therapy, low-dose IL-2-mediated Treg expansion, and antigen-specific Treg cellular products demonstrate promising efficacy in managing autoimmune disorders and preventing transplant rejection [55,78]. Additionally, monoclonal antibodies, small molecules, and cell-based therapies targeting tumor-infiltrating Tregs aim to augment antitumor immunity [79]. Recent advances in cell engineering, ex vivo expansion protocols, and antigen-specific targeting have facilitated early-phase clinical trials of Treg-based therapies, which demonstrate promising restoration of immune tolerance alongside favorable safety profiles [80].

However, substantial challenges impede the translation of Treg therapies into broad clinical practice. Maintaining the phenotypic stability, antigen specificity, and long-term persistence of infused Tregs in vivo remains technically demanding [81]. Furthermore, mitigating the risk of excessive immunosuppression potentially predisposing patients to opportunistic infections or oncogenesis, necessitates refined dosing strategies and rigorous monitoring protocols [13,82]. Additionally, substantial heterogeneity in Treg biology across diseases and among patients poses significant challenges to therapeutic implementation, necessitating meticulous optimization of isolation and expansion protocols [83]. Genetic, epigenetic, and environmental factors underlie interpatient variability in Treg function, abundance, and phenotype, resulting in heterogeneous suppressive capacity, stability, and therapeutic potential even among patients with identical diagnoses [71,84]. Overcoming these challenges through refined methodologies and rigorous clinical evaluation will be essential to unlocking the full therapeutic potential of Treg-based therapies.

## 9. Future Directions for Treg-Based Therapeutic Strategies

Future research directions and clinical strategies leveraging regulatory T cells are anticipated to revolutionize immunotherapy and disease management paradigms in the coming years. Advances in cellular engineering, including the generation of antigen-specific Tregs and the genetic enhancement of their suppressive capacity or persistence, may enable more precise and durable immunomodulatory strategies for autoimmune disorders, organ transplantation, and allergic diseases. However, the therapeutic potential and clinical applicability of these approaches remain under active investigation [85,86]. Emerging strategies to bolster Treg stability, suppressive potency, and targeted delivery to affected tissues incorporate advanced methodologies, including biomaterial scaffolds for localized retention, CRISPR/Cas9-mediated gene editing for functional enhancement, and combinatorial regimens integrating immune modulators to amplify therapeutic efficacy [21]. Transcriptomic and epigenetic profiling serves to validate and maintain the phenotypic stability and suppressive function of these genetically modified Tregs, enabling comprehensive quality control prior to clinical deployment. Integrating personalized and precision medicine paradigms into Treg-based therapies enables bespoke interventions tailored to individual patients’ immune profiles and disease-specific characteristics, thereby optimizing therapeutic efficacy while minimizing adverse effects [87,88]. Ongoing research endeavors will delineate how environmental cues, tissue-specific signals, and molecular determinants orchestrate Treg behavior, thereby informing next-generation therapeutic strategies. Innovations in single-cell omics, spatial transcriptomics, and gene-editing technologies (e.g., CRISPR/Cas9) are ushering in a transformative era for elucidating and engineering the context-specific functions of Tregs. Emerging therapeutic modalities seek to harness Treg suppressive activity with high precision, customizing interventions to discrete pathological milieus, such as autoimmunity, malignancy, chronic infections, and transplantation while mitigating risks of off-target immunosuppression [21,53,89]. Treg heterogeneity can shape therapeutic outcomes because different subsets are not equally suppressive, stable, or tissue-adapted; in cancer, some Treg states are linked to poor prognosis and resistance to immunotherapy, while others may be less pathogenic or context dependent. This means that selective targeting or expansion of the right Treg subset may improve treatment precision, whereas using mixed Treg populations can produce variable or unpredictable results [90]. Treg-based therapies have shown early clinical promise, especially in autoimmune and transplant settings, but their benefits are limited by challenges in persistence, phenotypic stability, and precise targeting. A major concern is that excessive or poorly controlled Treg activity may lead to unwanted immunosuppression, reducing host defense and potentially impairing anti-tumor immunity [91,92,93]. Rapid advancements in Treg biology have opened unprecedented opportunities to refine immunomodulatory strategies and improve clinical outcomes across a diverse array of immune-mediated diseases. The synergistic integration of personalized immunoprofiling with cutting-edge cell engineering techniques promises transformative, patient-specific Treg-based therapies poised to redefine disease management paradigms in the near future [94].

## 10. Conclusions

Regulatory T cells function as versatile guardians of immune homeostasis, exerting suppressive and reparative effects within specialized tissue microenvironments. Their influence is mediated through dynamic crosstalk with innate and adaptive immune compartments, employing diverse mechanisms including secretion of anti-inflammatory cytokines (IL-10, TGF-β, IL-35), CTLA-4-dependent modulation of antigen-presenting cells, and metabolic reprogramming via IL-2 consumption alongside adenosine production. Tregs employ multimodal suppressive mechanisms that enable dynamic adaptation to heterogeneous inflammatory cues, thereby orchestrating contextually precise regulation of immune responses. Notably, these cells acquire tissue-resident phenotypes and execute non-canonical functions, such as promoting tissue repair, regeneration, and metabolic homeostasis that are indispensable for physiological homeostasis and pathological resolution. Tregs preserve local and systemic immune homeostasis, thereby averting autoimmunity, curtailing pathological inflammation, and facilitating tissue integrity restoration after injury. Advancements in cell engineering, pharmacotherapy, and precision modulation have dramatically broadened the therapeutic horizon of Tregs. The convergence of innovative methodologies and improved mechanistic insights is advancing the development of Treg-based interventions, which are currently being evaluated for their potential applications in autoimmunity, malignancy, transplantation, and other immune-mediated disorders. The foremost challenge ahead lies in achieving precise regulation of Treg stability, plasticity, and tissue-specific functionality, while mitigating risks of over-immunosuppression, such as susceptibility to infections or inadvertently promoting immune escape in neoplastic contexts. In-depth interrogation of Treg molecular regulation and environmental adaptability will be pivotal to realizing their full therapeutic potential and devising sophisticated strategies for immune homeostasis restoration. Fundamentally, Tregs emerge as master regulators of immunological fitness, indispensable for upholding immune equilibrium and self-tolerance, while heralding transformative therapeutic prospects across a broad spectrum of immune-mediated pathologies.

## Data Availability

No new data were created or analyzed in this study. Data sharing is not applicable to this article.

## Abbreviations

The following abbreviations are used in this manuscript:

Tregs	Regulatory T cells
FOXP3	Forkhead box protein P3
IL	Interleukin
TGF-β	Transforming growth factor beta
APCs	Antigen presenting cells
CTLA-4	Cytotoxic T-lymphocyte associated protein-4
PD-1	Programmed cell death protein-1
TME	Tumor microenvironment
nTregs	Natural Treg cells
pTreg	Peripheral Treg cells
GITR	Glucocorticoid-induced tumor necrosis factor receptor-related protein
LAG-3	Lymphocyte activation gene-3
TIGIT	T cell immunoreceptor with Ig and ITIM domains
LAP	Latency-associated peptide
GARP	Glycoprotein A repetitions predominant
TNFR	Tumor necrosis factor receptor
RORγt	Retinoid-related orphan receptor gamma t
AREG	Amphiregulin
PPAR-γ	Peroxisome Proliferator-Activated Receptor gamma
IFN-γ	Interferon gamma
mTOR	Mammalian target of rapamycin
PI3K	Phosphatidylinositol 3-kinase
AKT	Protein Kinase B
GvHD	Graft-versus-host disease
CAR	Chimeric antigen receptor
DMARD	Disease-modifying antirheumatic drug
TNF-α	Tumor necrosis factor-alpha
CRISPR/Cas9	Clustered regularly interspaced short palindromic repeats and CRISPR-associated protein 9
Tregs	Regulatory T cells
FOXP3	Forkhead box protein P3
